# Esophageal Perforation by Fish Bone Ingestion Causing Purulent Pericarditis

**DOI:** 10.14309/crj.0000000000001291

**Published:** 2024-03-04

**Authors:** Tamara Arruda, Vinícius Nina, Nilo Souza Filho, Aubyn Marath, João Pedro Abreu

**Affiliations:** 1 Department of Cardiovascular Surgery, University Hospital of the Federal University of Maranhão/HU-UFMA, São Luís, Maranhão, Brazil; 2 Department of Thoracic Surgery, University Hospital of the Federal University of Maranhão/HU-UFMA, São Luís, Maranhão, Brazil; 3 CEO Cardiostart International, Eugene, OR; 4 Federal University of Maranhão, São Luís, Brazil

**Keywords:** Purulent pericarditis, Esophageal perforation, Thoracic surgery

## Abstract

A previously healthy 38-year-old woman presented with new-onset sudden chest pain radiating to the back, associated with cough, dyspnea, nausea, vomiting, and gastric fullness after eating a bony fish. A diagnosis of gastroesophageal reflux disease was made. After a week of progressive worsening of her symptoms, she was referred to the specialist hospital. There, computed tomography imaging strongly suggested that a likely fishbone had penetrated the esophagus into the mediastinal structures; it seemed to have produced a pneumopericardium. Other tests suggested diffuse changes in ventricular repolarization, pericardial thickening, and diastolic restriction. Exploratory thoracotomy confirmed esophageal-pericardial perforation by the fishbone and purulent pericarditis. Despite appropriate surgical repair, the patient died on fifth postoperative day from an asystolic cardiac arrest that was refractory to repeated attempts to resuscitate her.

## INTRODUCTION

Esophageal lesions, such as those caused by the ingestion of foreign bodies that breach the wall, are a very uncommon condition, but they represent a challenge to specialist health services because they may present difficulties in diagnosis, clinical management, and are associated with a high lethality.^1^ The anatomical relationship of the esophagus to several vital intrathoracic structures, especially the heart, makes the identification of the exact site of the lesion very important for a timely operative approach; it may directly impact the survival of this life-threatening injury.^2^

In this report, we present a case of a patient who was initially diagnosed with gastroesophageal reflux disease (GERD), but who developed symptoms not in keeping with the initial diagnosis. Subsequent investigation led to the diagnosis of purulent pericarditis caused by perforation of the esophagus and pericardium after ingestion of a fishbone. This report was performed following the CARE guidelines for case reports.^3^

## CASE REPORT

A previously healthy 38-year-old woman presented to a rural emergency department in a remote location 70 miles from a specialist center with sudden tight chest pain radiating to the back, associated with cough, dyspnea, nausea, vomiting, and gastric fullness. She had previously eaten a bony fish. That information may not have been emphasized, and an initial diagnosis of GERD was made. After 1 week, the patient's symptoms worsened, and she was locally hospitalized for a fresh and more detailed evaluation. These investigations led to the diagnosis of purulent pericarditis, which resulted in her referral to the tertiary care specialist hospital located in São Luís, Maranhão.

During admission to tertiary hospital, the patient's hemodynamic status was abnormal, with blood pressure of 100/70 mm Hg, heart rate of 136 bpm, and respiratory rate of 25 breaths per minute; she was distressed, had a persistent cough, and was increasingly dyspneic on mild exertion. Under sedation, orotracheal intubation was performed, and the patient then transferred to the cardiac intensive care unit and placed on mechanical ventilatory support. During this time, she had further investigations to establish a full diagnosis.

**Figure 1.**
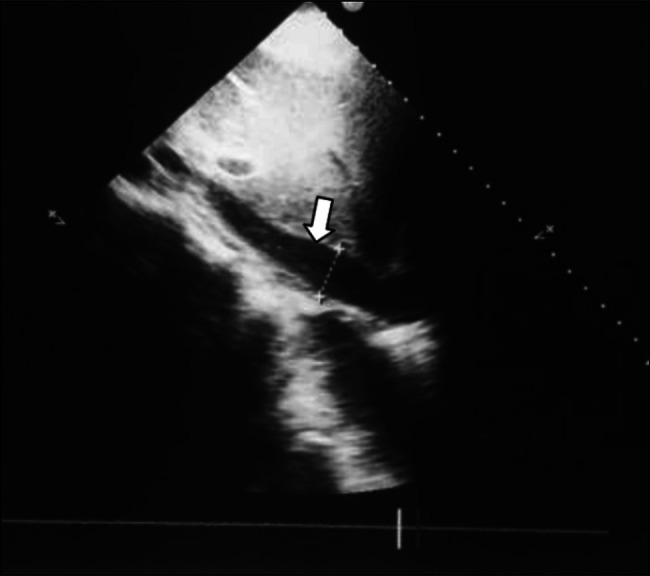
Echocardiogram showing thickening and pericardial effusion (white arrow).

**Figure 2.**
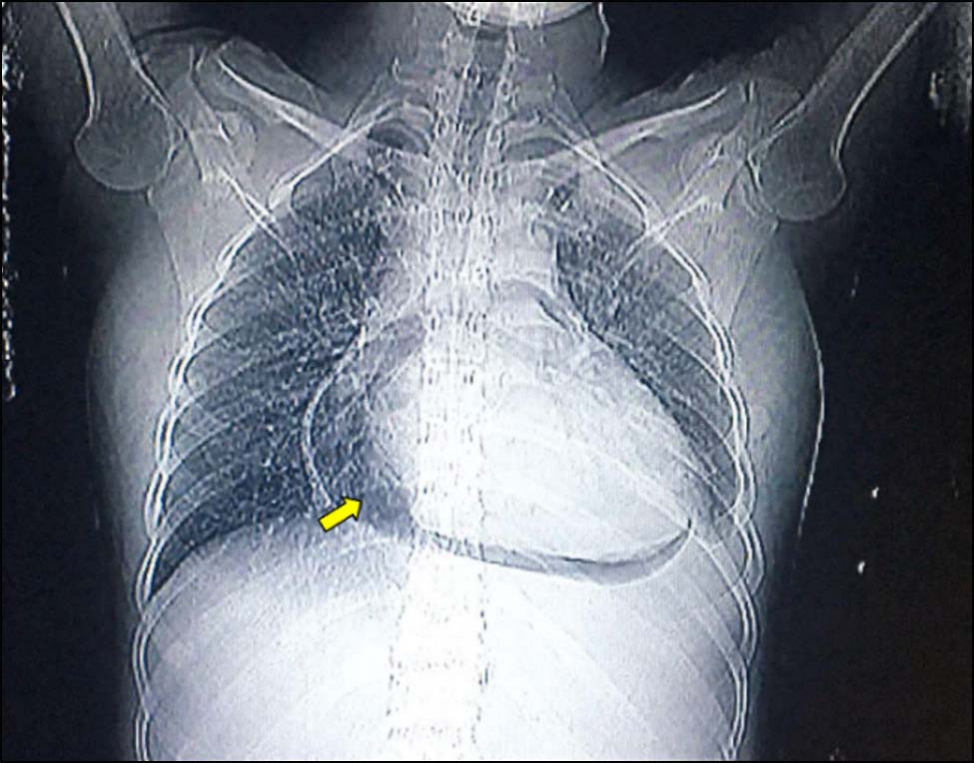
Chest X-ray showing pneumopericardium (yellow arrow).

**Figure 3.**
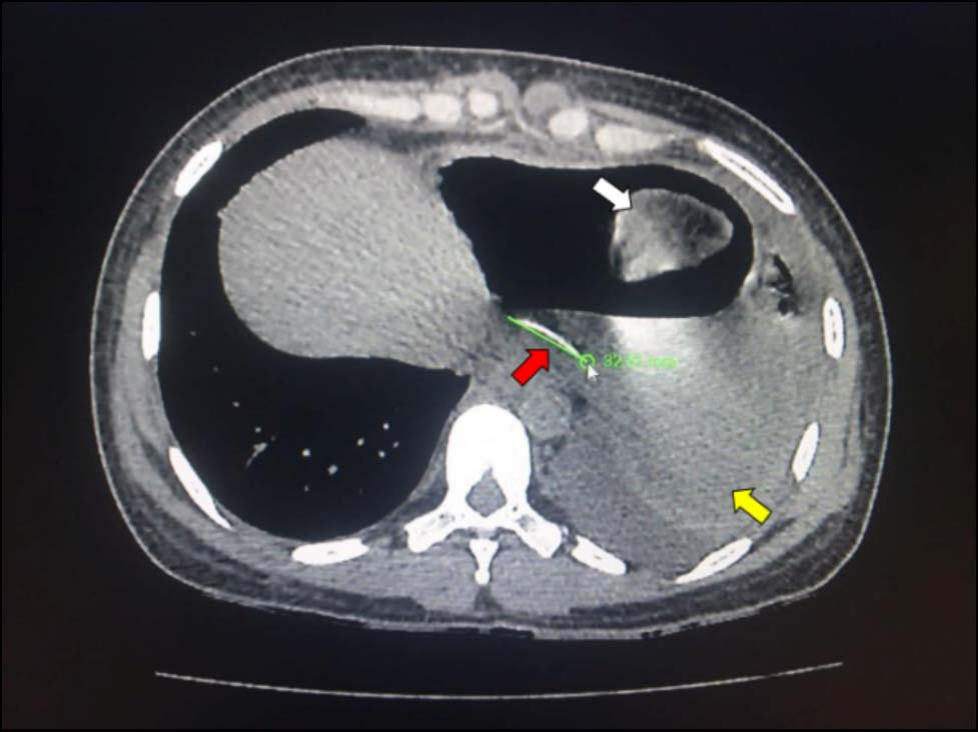
Chest CT showing fishbone measuring 3.4 cm (red arrow), pulmonary consolidation (white arrow), and pleural effusion (yellow arrow) on the left. CT, computed tomography.

Electrocardiography showed extensive anterior ECG ST segment elevation compatible with subepicardial injury, accompanied by morphological changes to the T wave. Transthoracic echocardiogram showed thickening of the pericardium with mild effusion and signs of diastolic restriction compatible with constrictive pericarditis (Figure 1). Chest X-ray showed a pneumopericardium; a chest CT scan showed the presence of a hyperdense artifact (suggestive of a fishbone) in the pericardial area measuring 3.4 cm, which was juxtaposed to a bulky pneumopericardium; it also revealed pulmonary consolidation and a left pleural effusion (Figures 2 and 3).

Laboratory tests showed a leukocytosis with elevated neutrophils count (22,350/mm³), hemoglobin = 9.9 g/dL, hematocrit = 29.9%, alanine transaminase = 150 U/L, and aspartate aminotransferase = 90 U/L, which were suggestive of an active infectious process. Antibiotic therapy with cephalothin and gentamicin was then initiated.

**Figure 4.**
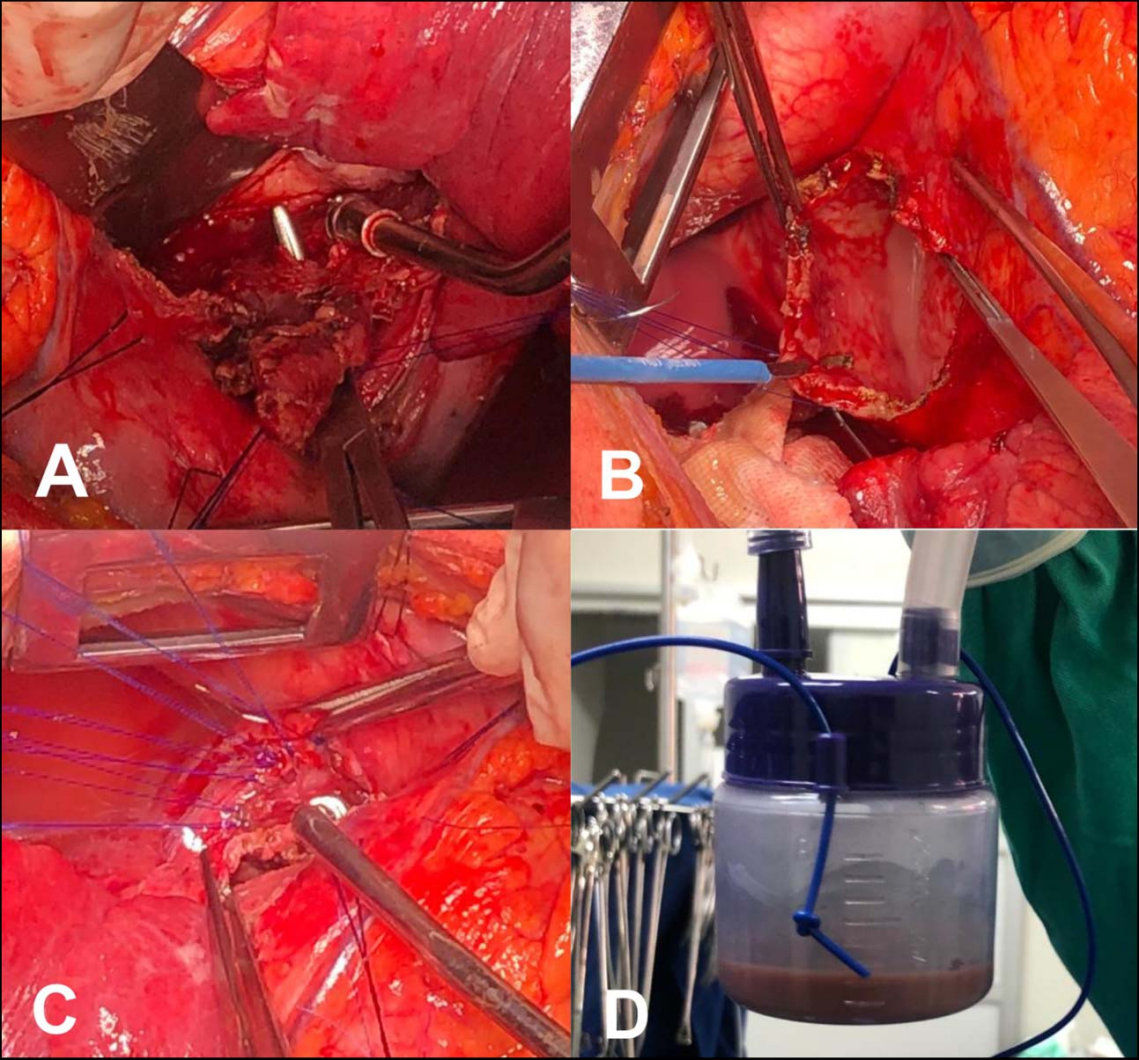
(A) Esophageal perforation. (B) Purulent pericardial effusion. (C) Esophageal perforation repair. (D) Sample of the pleuropericardial collection for microbiological analysis.

After a multidisciplinary team discussion, a left lateral thoracotomy in the fourth intercostal space was performed. An esophageal-pericardial communication was identified by instilling a solution containing methylene blue within the nasogastric tube (Figure 4). About 500 mL of fetid, purulent fluid was released on opening the pericardium. The pleuropericardial collection was washed out with saline solution, and the esophageal lesion was closed with separate 3-0 polypropylene stitches. A partial pericardiectomy was performed to reduce the constrictive effect. The operation was completed using a warm saline washout, and a standard closure technique included the insertion of 2 tubular underwater seal drains.

After thoracotomy, the patient's hemodynamic status worsened, and she developed tachycardia, reduced oxygen saturation, and respiratory acidosis. Mechanical ventilation was adjusted, resulting in some improvement in respiratory parameters the following day. Later, the patient's condition worsened, and she became oliguric, remained febrile, and was hemodynamically unstable at intervals. In trying to reverse her deteriorating trend, she was given noradrenaline, vasopressin, and dobutamine. Mechanical ventilation was maintained under analgesia with midazolam and fentanyl with appropriate adjustments as needed. During this period, the patient's mean arterial pressure ranged from 58 to 98 mm Hg, and heart rate ranged from 145 to 158 bpm. On the second postoperative day, the patient became anuric and more hemodynamically unstable, despite the infusion of vasoactive drugs. It was felt that she had now developed mixed shock (septic and cardiogenic), and she exhibited severe biventricular dysfunction and atrial fibrillation with a high ventricular rate response. Intravenous calcium replacement was given as needed, and hemodialysis was initiated. Microbiological tests from swabs taken within different operative sites revealed the growth of Gram-negative bacteria, *Burkholderia cepacia*, *Sphingomonas paucimobilis*, and *Klebsiella pneumoniae*. Antibiotic therapy was adjusted to meropenem and teicoplanin, with dose adjustments and regular review to consider her severely impaired renal function.

Despite intensive clinical management, the patient remained febrile, anuric, with leukocytes of 23,620 mm³, platelets of 10,200 mm³, hematocrit of 27.1%, hemoglobin of 8.9 g/dL, bilirubin of 2.88 mg/dL, international normalized ratio of 4.26, alanine transaminase of 654 μ/L, alkaline phosphatase of 4,088 μ/L, potassium of 6.7 mg/dL, urea of 189 mg/dL, creatinine of 5.7 mg/dL, and creatine kinase-MB of 252 ng/mL. The patient remained anuric, on hemodialysis, needing full ventilatory support on the fourth postoperative day; she subsequently developed diarrhea and visible areas of necrosis in the extremities with blistering. A progressive increase in the boundaries of these areas rapidly occurred. Regional amputation of the necrotic distal limb sections was considered as an emergency option to excise the necrotic field surgery, but on discussion with the family members, they refused to allow the procedure. They were appropriately advised and made aware of the complications and possible outcome of their decision. On the fifth postoperative day, further clinical and hemodynamic deterioration occurred, with agonic breathing and the need for an increasing dose of vasoactive drugs. Finally, the patient suffered a terminal cardiac arrest in asystole, with no return to spontaneous circulation, despite applying standard resuscitative efforts.

## DISCUSSION

Transesophageal foreign body perforation represents a rare but potentially fatal condition. In the United States, this type of injury results in approximately 1 in 100,000 visits per year to healthcare services.^1^ The typical presentation of this condition may be characterized by dysphagia of sudden onset, sialorrhea, pain in the throat and retrosternal region, foreign body sensation, nausea, vomiting, and, in cases of airway involvement, dyspnea.^1^ A patient's symptomatology may vary, however, because of the presence of several structures adjacent to the esophagus; involvement of any of these may then lead to mediastinitis and pericarditis.^2,4^

In our report, the patient sought the rural health service's advice after presenting the aforementioned symptoms but received the initial diagnosis of GERD. Because it is a rare condition, esophageal perforations by foreign bodies may not be appreciated initially, and this may, of course, lead to an incorrect diagnosis and management.^5^ Of paramount importance, primary evaluation of sudden-onset dysphagia with any other symptoms should include obtaining the patient's history in depth, physical and biochemical examination, and be accompanied by early imaging tests that include radiography and computed tomography (CT) scan.

CT is also vitally important to determine the location, shape, and size of the foreign body and its proximity to other vital structures. It may also assist the evaluation of complications related to this condition, such as perforation of attached structures, abscesses, mediastinitis, and aortic fistulas.^1,5–8^

We used a multidisciplinary consultative approach to evaluate the patient's clinical presentation and reached a consensus for an emergency exploratory left lateral thoracotomy to be performed. Such early intervention is strongly recommended by other reports of such critical injuries.^4,9,10^ In cases of esophageal injury, the reinforcement of the suture line using an intercostal muscle or pleural flap may be considered to prevent dehiscence; however, because of advanced mediastinitis and consequently the high risk of spreading the infection to the chest wall, we decided to use biological adhesive only with no signs of suture failure postoperatively.^11^

The delay in the initial diagnosis of esophageal perforation by the fishbone may have been decisive in inflicting the demise of our patient. The mortality reported in the literature for those patients with esophageal perforations addressed within 24 hours ranges from 10% to 25%, contrasting with 40% to 60% in those patients whose surgical intervention is performed more than 24 hours after the injury.^6,9,12^ In our general experience, patients in rural settings in Brazil do sometimes present late with various clinical conditions because of the limited resources and lack of locally available healthcare workers. In some, the very large distances from patients home to a tertiary referral center may present severe challenges and delay a patient's transfer and subsequent review; in this case, it may have been a critically important negative, contributing factor to her clinical deterioration to sepsis and multiorgan failure.

Imaging studies, which include CT scan, should be performed at the earliest opportunity and, with the aforementioned symptomatology, performed within the first 24 hours whenever possible. With the advantage of early diagnosis and appropriate clinical management, it may then be possible to avoid sepsis and other complications. For esophageal lesions caused by foreign body perforation, careful attention to the patient's historical account and the absence of any previous dysphagia or related symptoms may offer vital clues to investigating, confirming the diagnosis of likely perforation and dealing with it surgically.

## DISCLOSURES

Author contributions: T. Arruda: substantial contributions to the conception, acquisition, analysis, design, or interpretation of data for the work. V. Nina and N. Souza Filho: drafting the work and reviewing it critically for important intellectual content. A. Marath and JP Abreu: reviewing the work critically for intellectual content. T. Arruda is the article guarantor.

Financial disclosure: None to report.

Informed consent was obtained for this case report.
